# Post-Exertional Malaise Is Associated with Hypermetabolism, Hypoacetylation and Purine Metabolism Deregulation in ME/CFS Cases

**DOI:** 10.3390/diagnostics9030070

**Published:** 2019-07-04

**Authors:** Neil R. McGregor, Christopher W. Armstrong, Donald P. Lewis, Paul R. Gooley

**Affiliations:** 1 Faculty of Medicine, Dentistry and Health Sciences, University of Melbourne, Parkville VIC 3010, Australia; 2 Department of Biochemistry and Molecular Biology, Bio21 Molecular Science and Biochemistry Institute, 30 Flemington Road, Parkville VIC 3010, Australia; 3 CFS Discovery, Donvale Medical Centre, Donvale VIC 3111, Australia

**Keywords:** fatigue syndrome, chronic, exercise, hypoacetylation, methylhistidine, histone deacetylation

## Abstract

Post-exertional malaise (PEM) is a cardinal predictive symptom in the definition of Myalgic Encephalomyelitis/Chronic Fatigue Syndrome (ME/CFS). If the cases overexert themselves they have what is termed “payback” resulting in a worsening of symptoms or relapse which can last for days, weeks or even months. The aim was to assess the changes in biochemistry associated with the cases self-reported PEM scores over a 7-day period and the frequency of reporting over a 12-month period. Forty-seven ME/CFS cases and age/sex-matched controls had a clinical examination, completed questionnaires; were subjected to standard serum biochemistry; had their serum and urine metabolomes analyzed in an observational study. Thirty-five of the 46 ME/CFS cases reported PEM in the last 7-days and these were allocated to the PEM group. The principal biochemical change related to the 7-day severity of PEM was the fall in the purine metabolite, hypoxanthine. This decrease correlated with alterations in the glucose:lactate ratio highly suggestive of a glycolytic anomaly. Increased excretion of urine metabolites within the 7-day response period indicated a hypermetabolic event was occurring. Increases in urine excretion of methylhistidine (muscle protein degradation), mannitol (intestinal barrier deregulation) and acetate were noted with the hypermetabolic event. These data indicate hypoacetylation was occurring, which may also be related to deregulation of multiple cytoplasmic enzymes and DNA histone regulation. These findings suggest the primary events associated with PEM were due to hypoacetylation and metabolite loss during the acute PEM response.

## 1. Introduction

Myalgic Encephalomyelitis/Chronic Fatigue Syndrome (ME/CFS) is a medically unexplained condition which occurs predominantly in females. It is characterized by persistent or relapsing fatigue and altered responses to exercise and alterations in normal sleep structure. Post-exertional malaise (PEM) is 10.4-fold more frequent in ME/CFS cases compared with controls [1]. However, very little is known about the underlying pathophysiology of PEM.

ME/CFS females were reported to have biochemical changes consistent with the deregulation of glycolysis and urea cycle activity [2], which were indicated by increases in the fasted first morning serum metabolome glucose and falls in lactate and acetate. The deregulation of glycolysis at pyruvate dehydrogenase (EC 1.2.4.1), has been confirmed by other researchers [3]. This deregulation of glycolysis results in falls of acetate and activation of histone deacetylation [4,5] as well as deregulation of acetylation of cytoplasmic and mitochondrial enzymes. Importantly, histone deacetylase 2 (HDAC2) was ~four fold higher and HDAC3 was ~two-fold higher, in ME/CFS cases compared with controls [6]. In further support, a study of gene upregulation in ME/CFS cases, following an exercise test, revealed two histone genes were upregulated [7]. Analysis of HDAC binding sites within the genes of that study revealed that 19 of the 20 upregulated genes had binding sites for HDAC1 and HDAC2 but also members of the SMAD transcription factor family that convey the signal from the transforming growth factor beta (TGF-β) receptor, namely SMAD1, SMAD4, and SMAD5 [8,9] (Table S1). The Whistler et al. study [7] also supports the hypothesis that acetylation changes may occur, when ME/CFS cases have PEM. Anomalies in TGF-β have also been identified in some ME/CFS studies but not all [10,11]. However, none of these were assessed against PEM activity. These data indicate that the change in glycolysis in ME/CFS cases may be related to either or combined effects of at least: (1) histone deacetylation; (2) a chronic reduction in acetate production via glycolysis; (3) deregulation of cytoplasmic and mitochondrial enzyme acetylation.

Reductions in the purine metabolite, hypoxanthine, were also found in the serum metabolomes of the females in first morning fasted samples [2] and potentially indicated reductions in the ability to produce ATP. During exercise, the release of hypoxanthine from muscle occurs as part of a hypermetabolic event when the levels of mitochondrial/cytoplasmic ATP fall. The hypermetabolic event relates to the release of metabolites from muscle associated with inhibition of protein synthesis within muscle once exercise starts. This same event occurs in lymphocytes when glycolysis is inhibited [12,13]. Whilst multiple immune issues have been detected in ME/CFS cases, the underlying mechanism behind the changes have not been identified [14]. Activation of glycolysis and histone acetylation are essential steps in immune activation [15], in particular T-cells and NK-cells [16]. An interesting study of lymphocytes showed that when the ATP levels fell after inhibition of glycolysis and adenosine degradation products increased, the incorporation of leucine into protein was also dramatically inhibited [12]. Thus, the ME/CFS case immune system issues may be a result of glycolytic and acetylation dysregulation, resulting in a reduced ability to translate DNA into proteins and hence protein synthesis. Evidence also indicates a switch toward utilization of branched-chain amino acids as an energy source, especially during exhaustive events [17].

Acetate is associated with control of multiple enzymes within the cell [18], which could be critically important in the biochemical changes in ME/CFS. A total of 1750 cellular proteins have been identified to have the characteristics to bind acetate and alter the protein function, these include DNA replication (52 proteins), DNA repair (72 proteins), cell cycle switching (132 proteins), nucleotide exchange factors (55 proteins), and acetylation and deacetylation (21 proteins) [18]. The biochemistry of these acetate regulated events may be secondary to the fall in acetate but are likely to have profound effects upon cellular function in ME/CFS cases.

The objective of this paper was to assess PEM 7-day severity and 12-month frequency symptoms scores and related biochemistry (blood and urine) in ME/CFS cases and controls. Associations of the PEM scores were examined using standard serum biochemistry, a 24-hour urine assessment and a blood and urine metabolome.

## 2. Methods

Forty-six cases with ME/CFS and 26 fatigue-free, age and sex-matched healthy individuals were recruited. Obtaining age and sex-matched controls was undertaken by placing advertisements on University billboards and selecting individuals of the same sex and age (±5 years) who were then assessed using the same clinical examinations. The ME/CFS group comprised cases that were currently symptomatic and diagnosed as having ME/CFS in accordance with the Canadian guidelines and its exclusionary criteria [19]. A Depression Anxiety Stress assessment (DASS) [20] was used to assess their psychiatric comorbidity. Only those ME/CFS subjects who complied with the criteria were included in the study. All subjects were asked to list their drugs and oral supplements. None of the participants were related to one another nor did they ever live together. All subjects signed consent forms. This study was approved by the University of Melbourne human research ethics committee (HREC# 0723086, 2010) and the first specimens were collected on 23 September 2010.

### 2.1. Clinical Measures

The subjects had a full clinical examination and were questioned about their illness, onset, and family histories to determine whether they fulfilled criteria for the Canadian ME/CFS guidelines. All subjects completed several questionnaires, including a large symptom questionnaire developed for chronic pain research [21]. It asked subjects to score how severe a symptom was in the last seven days (0–4 scalar response) and how frequently the symptom occurred over the last 12-month period (0–4 scalar response), as previously published [22]. The 7-day severity and 12-month frequency scores, designed to differentiate between acute and chronic responses, have been assessed against the biochemistry and found to differentiate between an acute response and a chronic response [22]. These questionnaires were checked for completeness by the reception staff and the medical clinician (DL). The medical clinician questioned the subjects about their responses to ensure accuracy.

### 2.2. Biochemistry Assessments

The sample collection and processing has previously been published [2] and are provided here in summary. Subjects had a phlebotomy for either standard serum biochemistry, which was performed at a commercial (National Association of Testing Authorities (NATA) accredited) laboratory; or a metabolome. A second urine sample was collected upon rising by each subject at their home and stored at 4 °C. Within 6 h, a blood sample was taken by venipuncture into BD Vacutainer® blood collection tubes (Beckton Dickinson, Mississauga, ON, Canada). All samples were stored at −80 °C prior to performing an NMR analysis as previously described [2] using a liquid-liquid extraction technique [23], data were acquired on an 800 MHz Bruker Avance II NMR spectrometer (Bruker, Billerica, MA, USA); metabolites were identified and quantitated with the compound libraries in the Chenomx software (v6.1, Chenomx, Edmonton, AB, Canada). Twenty-nine metabolites per blood serum sample and thirty metabolites per urine sample were identified.

### 2.3. Data Analysis

The metabolome data were prepared as raw data (µM) and as relatively distributed data (%) by dividing each metabolite concentration by the total concentration of metabolites quantified in each sample. The parametric data prior to statistical analysis was assessed for normality and log converted if not normally distributed. All percentage data were arcsine converted for analysis. The dataset was evaluated using Statistica for Windows Ver. 12.0 (TIBCO Software, Palo Alto, CA, USA) using statistical calculations, including t-tests and Pearson correlation coefficients, ANOVA and multivariate analysis. The nonparametric data were assessed using Spearman rank correlations, Mann-Whitney U-tests or Kruskal-Wallis ANOVA. Small sample frequency data were analyzed with Fisher exact Chi-square analysis (χ^2^). Multiplicity (Bonferroni) correction was carried out on the data based upon the number of variables assessed in each statistical test.

## 3. Results

### 3.1. Demographics

To examine the PEM biochemistry, we chose to divide the ME/CFS group on presence/absence of significant PEM responses in the last 7 days (PEM, NoPEM meaning those without current symptoms, Controls (C)). Table 1 shows the subject demographics for the ME/CFS cases divided on the basis of the presence or absence of significant PEM and the control group. No differences were found for sudden versus gradual onset or triggers at onset, such as infections (significance *p* < 0.01). Table 1 also shows the ME/CFS-defined symptom clusters across the three groups (control, ME/CFS: NoPEM, PEM). Apart from the PEM scores, there were no differences in symptom profiles between the NoPEM and PEM groups. Both groups presented higher scores compared with controls.

### 3.2. Biochemistry

Table 2 shows a summary of the statistically significant metabolome measures between the groups. The ME/CFS groups had significant reductions in serum hypoxanthine (NoPEM 4.4-fold lower, PEM 2.4-fold lower versus controls), serum lactate (NoPEM 1.9-fold lower, PEM 1.6-fold lower versus controls), phenylalanine (Both NoPEM and PEM 1.3-fold lower versus controls). Glucose was increased in the ME/CFS cases (both NoPEM and PEM 1.2-fold higher versus controls). In the urine the fall in acetate was greatest (NoPEM 2.5-fold lower, PEM 1.5-fold lower versus controls) and this was statistically different between the NoPEM and PEM subgroups (*p* < 0.01). The excretion of methylhistidine was higher in the PEM subgroup (1.6 fold) and control (1.3 fold) groups, respectively, compared with the NoPEM subgroup. In the fecal metabolome, the % butyrate was increased in the NoPEM group compared with both the PEM and control groups. (Figure S1 shows the group canonical plots of separations using different analyses).

Table 3 is a summary of the correlation analysis of the PEM scores across the whole group and within the ME/CFS group. In the whole group analysis, the 7-day severity PEM score and 12-month frequency PEM scores were positively correlated with serum glucose and negatively correlated with hypoxanthine, phenylalanine, lactate and threonine. No significant correlates were noted within the ME/CFS group. The absolute urine levels showed a significant correlation between the 7-day PEM score along with mannitol, serine, acetate, methylhistidine and glucose. The 12-month frequency of PEM correlated with a fall in acetate alone. The urine percentage data showed falls in urea, pyruvate and acetate with both the 7-day severity and 12-month frequency scores. The only fecal component to reach statistical significance was the percentage uracil. These data show a significant renal concentrating issue is occurring in the ME/CFS group during a PEM event and this was principally related to falls in urea and acetate. To check this, we calculated the serum to urine ratios of multiple metabolites. The 7-day severity of PEM correlated with the following ratios: serum acetate:urine acetate ratio (*r* = −0.44, *p* < 0.002), serum tyrosine:urine tyrosine ratio (*r* = −0.40, *p* < 0.006), serum serine:urine serine ratio (*r* = −0.39, *p* < 0.008), serum creatine:urine creatine ratio (*r* = −0.38, *p* < 0.009), and the serum leucine:urine leucine ratio (*r* = −0.37, *p* < 0.01). Thus, the 7-day severity of PEM was associated with an increased urinary excretion of metabolites within the ME/CFS group and this was associated with a reduction in multiple serum metabolites including the important protein synthesis regulating amino acid, leucine.

The serum glucose:lactate ratio is very similar to the changes in the urine glucose:lactate ratio, which is consistent with the change seen in the serum acetate: urine acetate ratio. This suggests that the serum and urine changes are very similar. Thus, the available acetate in serum appears to be significantly reduced and is lowest in the NoPEM cases.

### 3.3. Purine Metabolism Changes

As serum hypoxanthine was the prime predictive variable for alterations in the PEM scores, we assessed the relationships between serum Hypoxanthine and the purine related metabolites (Table 4). Serum and urine hypoxanthines were lower in the PEM subgroups versus the controls. Whilst there was no difference in the serum urate levels, the marker of purine degradation in the liver, the serum hypoxanthine: urate ratio was lower in the ME/CFS group. The ratio in the NoPEM subgroup was 5.4-fold lower whilst in the PEM group the ratio was 3.5-fold lower. The hypoxanthine:urate ratio was negatively correlated with serum glucose (*r* = −0.48, *p* < 0.001) and positively correlated with serum lactate (*r* = 0.77, *p* < 0.001), the purine ring precursor amino acids (*r* = 0.54, *p* < 0.001), acetate (*r* = 0.49, *p* < 0.001), and the total serum amino acids (*r* = 0.38, *p* < 0.006). The correlation between serum hypoxanthine and the purine ring precursors, indicative of purine synthesis, was not different between the ME/CFS cases and the controls (ME/CFS *r* = 0.66, *p* < 0.001, control *r* = 0.61, *p* < 0.001). However, the ratio was significantly lower in the ME/CFS group (Table 4 and Figure S2) and the purine ring precursor amino acids correlated positively with serum acetate (*r* = 0.52, *p* < 0.001). Thus, the synthesis and possibly the salvage of hypoxanthine were reduced whilst purine degradation was in the normal range. The levels of hypoxanthine in the serum were associated with the availability of the purine ring precursors, the glucose: lactate ratio and acetate. This suggests that acetylation is a major factor in the change in the purine metabolism deregulation in ME/CFS. Thus, the increase in urine metabolite loss during exercise events in ME/CFS cases results in a loss of purine ring precursors and a fall in acetate and hypoxanthine.

## 4. Discussion

This paper has identified that the post-exertional malaise experienced by an Australian Anglo-Celtic cohort of ME/CFS cases is associated with a deregulation of purine metabolism and low acetate levels. This deregulation of purine metabolism is associated with a change in glycolytic activity and a switch to urea cycle creatine phosphate energy usage [2]. This has the effect of reducing the availability of acetate and upregulating histone deacetylase activity [4]. A four- and two-fold increase in HDAC2 and HDAC3, respectively, have been confirmed in ME/CFS cases [6] and a very high level of HDAC1 and HDAC2 binding sites occur within the genes upregulated in ME/CFS cases following exercise (see Table S1) [7]. The enzyme hypoxanthine phosphoribosyltransferase (EC 2.4.2.8) is an important enzyme in the salvage of the purines, adenosine and guanine [24]. Its gene (*HPRT1*) is on the X chromosome and has an unusual regulatory issue. Acetylation and methylation of one X chromosome silence its activity in females, which results in a single X chromosome being active for transcription, as in males [25,26]. This potentially poses a significant issue if there is a loss of silencing of the second X chromosome. This study has too few males to properly assess this potential issue. Deregulation of X chromosome silencing may be related to the fall in hypoxanthine salvage and the more severe illness in females compared with males [1]. Studies are warranted to investigate this interesting possibility.

There is increased urine excretion of metabolites associated with the 7-day PEM scores, in-particular, mannitol, methylhistidine, acetate, and glucose. This increased metabolite excretion correlates with the ME/CFS case reported 7-day severity of PEM symptoms. Relative abundance assessment shows that the efflux of metabolites is associated with reductions in urine urea, pyruvate and acetate suggesting an energy and renal concentrating issue, possibly associated with hypoacetylation, is most likely occurring at the time of the metabolite loss. In diabetic nephropathy, the renal tubular cells upregulate glycolysis and lactate production [27]. This may also be the case in this study as the excretion of glucose (*r* = +0.37, *p* < 0.01) and acetate (*r* = +0.41, *p* < 0.01) were positively correlated with the 7-day PEM severity. Importantly, acetate was negatively correlated with the 12-month frequency of PEM events (*r* = −0.32, *p* < 0.01). This change in renal acetate retention is also supported by the negative correlation between the 7-day PEM score and the serum acetate:urine acetate ratio (*r* = −0.44, *p* < 0.002). Thus, the greater the frequency of PEM events the greater the loss of acetate. Renal glomerular podocytes are damaged by increases in blood glucose in diabetic patients and this has been linked to deacetylation of Nephrin and microRNA activity [28]. In this study, the reduction in serum acetate levels appears to result in a conditional renal hypoacetylation event which will allow increased metabolite loss from the kidney. Renal changes in diabetes nephropathy are also associated with down-regulation of bone morphogenic protein (BMP) receptor function and TGF-β mediated transcription factor production and supply of BMP-7 restores function [29,30]. Whilst the renal changes are very similar to the renal changes seen in central diabetes insipidus, protein-calorie restriction and infection/inflammatory mediated events, no subjects had diabetes insipidus or were protein calorie restricted, and all had average BMI’s. These renal changes provide additional support for either an inflammatory origin or a possibly an energy/acetylation or even a transcription factor problem. Importantly, multiple studies have found that the level of serum cytokines are not significantly different between ME/CFS and controls and do not correlate with symptom expression [31]. Therefore, studies to assess the activities of HDAC and BMP transcription factors in MEC/CFS cases are warranted.

The change in renal metabolite loss is associated with increased mannitol excretion, which suggests a gastrointestinal barrier issue may also be occurring. NoPEM ME/CFS cases had a 3.2-fold lower urinary mannitol, not unlike that seen in multiple sclerosis patients [32]. However, the level of urinary mannitol increased with the 7-day PEM scores (*r* = +0.38, *p* < 0.01). This increase in mannitol indicates a potential intestinal barrier change, which is consistent with the finding of bacteremia following exercise in ME/CFS cases [33]. The presence of bacteremia is supported by the correlation between fecal uracil and the 7-day PEM score (*r* = +0.46, *p* < 0.001). The increase in fecal uracil was also correlated with the serum hypoxanthine level in the PEM group (*r* = +0.39, *p* < 0.03) showing that they rose together as part of the PEM-associated hypermetabolic event. Uracil is a breakdown product of RNA but may also be of bacterial origin. Whether this indicates a breakdown in enterocytes or an alteration in the fecal flora or their metabotoxins/toxins is not known. Further investigation of these changes is warranted.

A 1.6-fold increase in urinary excretion of methylhistidine within the PEM subgroup was also seen compared with the NoPEM subgroup. Methylhistidine is a breakdown product of muscle contractile proteins, following a short term bout of resistance exercise [34]. Muscle protein synthesis is controlled by the available leucine and phenylalanine [35] and by BMP protein receptor activity [36]. In this study urinary methylhistidine positively correlated with urinary creatine (*r* = +0.63, *p* < 0.001), leucine (*r* = +0.59, *p* < 0.001), phenylalanine (*r* = +0.40, *p* < 0.001) and acetate (*r* = +0.47, *p* < 0.001) across all groups. A reduction in available acetate during exercise is associated with a reduction in phosphocreatine degradation and hence is associated with increased phosphocreatine and mitochondrial energy provision [37], which is consistent with the glycolysis/urea cycle energy switch identified in this ME/CFS cohort [2]. Interestingly, 3-methylhistidine in the nonacetylated form is excreted in greater amounts when rats are exposed to bacterial lipopolysaccharides [38]. It is likely that the increased 3-methylhistidine excretion observed during the 7-day PEM response is the result of the reduced energy provision and the fall in amino acids, which may be acetylation mediated. However, the response could also be exacerbated by the gastrointestinal barrier anomalies suggested by the increased bacteremia identified in ME/CFS cases [33]. Alternatively, an anomaly in BMP regulation may also be involved in the increased 3-methylhistidine excretion. Thus, a combination of at least three different events may contribute to the increased 3-methylhistidine excretion and this may be reflected in different genetic susceptibilities within different subjects.

The findings that the PEM is associated with a loss of metabolites, reduction in acetylation, deregulation of purine metabolism, increased contractile protein breakdown and bacteremia associated with exercise suggest that treatments such as graded exercise may be more detrimental than beneficial as claimed in some studies [39,40]. Until such time as these biological changes can be further investigated, the use of graded exercise as a therapy for those with severe forms of ME/CFS should be considered potentially harmful. In support of this, the use of graded exercise therapy has caused significant protest by ME/CFS sufferers as they see it as harmful [41,42].

This study was designed to investigate metabolic changes in ME/CFS subjects using a discovery hypothesis and not a specific hypothesis-driven method to assess specific biochemical events. This study with these limitations has resulted in the development of a hypothesis which now requires to be assessed by a typical hypothesis-driven process. Whilst the study size is small it reproduced the earlier findings but should be reproduced with a larger sample or multi-centers to reconfirm the findings. The use of self-reported symptoms may introduce a recall bias within the subjects and in a larger study, each of the variables found to be associated with the symptom severity and distribution need to be evaluated by other methods. Studies investigating acetylation and its related DNA transcription changes and the alteration in cytosol enzyme activity should allow the development of the understanding of the mechanisms of PEM development and the development of appropriate therapies based upon the underlying biochemistry.

## 5. Conclusions

This study revealed that post-exertional malaise is associated with changes in glycolysis and acetylation in ME/CFS cases. These changes are consistent with a hypoacetylation state and are likely to significantly alter histone acetylation and the actions of acetylation and deacetylation in controlling cellular enzymatic events. Well-designed studies evaluating these important factors are warranted.

## Figures and Tables

**Table 1:** Subject demographics, post-exertional malaise (PEM) scores within the ME/CFS cases divided on the basis of PEM 7-day severity scores compared with controls. The symptom scores are given as the median and the 10–90% percentile distribution.

	Control	ME/CFS NoPEM	ME/CFS PEM
Number	25	11	35
Age	33.6 ± 7.8 *	30.9 ± 9.6	42.1 ± 16.3
%Females	96%	100%	80%
Duration (years)	-	8.7 ± 5.4	12.1 ± 9.7
Age at Onset	-	22.8 ± 6.8	30 ± 13.9
Systolic BP	-	116 ± 12	129 ± 17
Diastolic BP	-	79 ± 7	83 ± 9
Pulse rate	-	78 ± 11	73 ± 13
BMI *x* ± SD	23.1 ± 2.6	22.7 ± 3.6	24.9 ± 6.1
DASS Depression	0	11.2 ± 10.7	11.1 ± 11.1
DASS Anxiety	0.5 ± 0.6	13.3 ± 8.3	9.8 ± 7.8
DASS Stress	3.5 ± 3.6 *	20.7 ± 9.4 *	12.9 ± 8.7
PEM 7D	0.1 ± 0.4 **	1.3 ± 0.65 **	3.7 ± 0.5 **^,‡^
12F	0.1 ± 0.4 **	2.8 ± 1.2 **	3.5 ± 0.6 **^,†^
Fatigue 7D	0.7 ± 0.8 **	3.2 ± 0.9 **	3.7 ± 0.6 **
12F	1.1 ± 0.8 **	3.8 ± 0.6 **	3.8 ± 0.4 **
Sleep Disturbance 7D	1.6 ± 2.6 **	10.9 ± 2.5 **	10.5 ± 3.2 **
12F	2.6 ± 2.3 **	12.3 ± 2.7 **	11.1 ± 2.8 **
Cognition scores 7D	2.3 ± 2.9 **	16.1 ± 5.8 **	17.3 ± 6.5 **
12F	3.6 ± 3.7 **	18.8 ± 5.6 **	18.3 ± 5.9 **
Body Pain 7D	1.3 ± 1.3 **	6.3 ± 2.1 **	6.3 ± 2.2 **
Distribution 12F	2.6 ± 1.3 **	7.1 ± 1.5 **	6.9 ± 2.1 **

Statistical methods: ANOVA and post-hoc Tukey Honest Significance (THS) analysis. Multiplicity (Bonferroni) correction: Data only included if one measure reached *p* < 0.01. PEM = PEM group. NoPEM = those with very low or absent PEM scores. BMI = body mass index, 7D = 7-Day severity score, 12F = 12-month frequency score. In control column * = *p* < 0.01 or ** = *p* < 0.001 for the ANOVA. In the ME/CFS NoPEM or PEM columns * = *p* < 0.01 or ** = *p* < 0.001 for post hoc analysis vs. controls. In the ME/CFS NoPEM or PEM columns † = *p* < 0.01 or ‡ = *p* < 0.001 for post hoc analysis of NoPEM vs. PEM.

**Table 2:** Summary of ANOVA assessment of the changes in the serum, urine and fecal metabolomes.

Serum	Control Mean (SD)	ME/CFS NoPEM Mean (SD)	ME/CFS PEM Mean (SD)
Hypoxanthine (μM)	15.7 ± 12.2 **	3.6 ± 1.4 **	6.6 ± 8.2 **
Lactate (μM)	637 ± 335 **	339 ± 68 *	399 ± 240 **
Phenylalanine (μM)	18.4 ± 3.1 **	15.5 ± 2.2	15.1 ± 3.5 **
Glucose (μM)	971 ± 233 *	1266 ± 249 *	1189 ± 318 *
Hypoxanthine %	0.55 ± 0.39 **	0.14 ± 0.05 **	0.24 ± 0.25 **
Lactate %	22.7 ± 10.5 **	12.9 ± 2.0 *	14.7 ± 6.5 **
Phenylalanine %	0.68 ± 0.09 **	0.60 ± 0.10	0.58 ± 0.10 **
Glucose %	36.2 ± 9.5 **	48.1 ± 5.3 **	45.4 ± 7.5 **
**Urine**			
Acetate (μM)	91.9 ± 60.3 **	37.0 ± 14.9 **	63.3 ± 31.8 ^†^
Formate (μM)	81.1 ± 56.1 *	27.1 ± 15.2 **	43.0 ± 30.3 *
Urea (μM)	7969 ± 3050 *	4868 ± 2678 **	5821 ± 2425
Mannitol (μM)	312 ± 198 *	96 ± 57 **	258 ± 344 *
Serine (μM)	383 ± 198 *	178 ± 108 **	313 ± 193
Pyruvate (μM)	22.2 ± 10.7 *	11.3 ± 6.4 **	18.4 ± 12.6
Hippurate (μM)	632 ± 424 *	297 ± 253 *	666 ± 612
Methylhistidine (μM)	278 ± 192 *	230 ± 418 *	358 ± 373 ^†^
Pyruvate %	0.36 ± 0.08 **	0.26 ± 0.11 *	0.27 ± 0.09 **
Urea %	4.7 ± 3.4 **	3.8 ± 5.3	5.6 ± 5.1 **
Serine %	6.1 ± 1.6 *	4.2 ± 0.9 *	4.8 ± 1.9 *
Creatinine %	19.7 ± 10.3 *	33.4 ± 9.2 *	25.7 ± 11.5
Acetate %	1.53 ± 0.67 *	0.94 ± 0.26 *	1.08 ± 0.61 *
Allantoin %	0.53 ± 0.28 *	0.96 ± 0.33 *	0.78 ± 0.53
Tryptophan %	0.49 ± 0.16 *	0.49 ± 0.35	0.36 ± 0.10 *
**Fecal**			
Butyrate %	9.8 ± 3.5 *	15.2 ± 4.5 *	11.3 ± 4.4 ^†^
**Ratios**			
Serum Glucose: Lactate	2.2 ± 1.4 **	3.8 ± 0.7 **	3.6 ± 1.4 **
Urine Glucose: Lactate	5.2 ± 2.3 *	7.8 ± 4.7 *	6.2 ± 1.8
Serum Glucose: Acetate	96.4 ± 53.6 **	150.6 ± 45.8 *	155.2 ± 72.0 **
Urine Glucose: Acetate	1.37 ± 0.61 *	1.91 ± 0.52 *	1.78 ± 0.70
Serum Acetate: Urine Acetate	0.16 ± 0.09 *	0.30 ± 0.18 *	0.18 ± 0.09 †

Statistical method: ANOVA, multiplicity correction *p* < 0.01. In control column * = *p* < 0.01 or ** = *p* < 0.001 for the ANOVA. In the ME/CFS NoPEM or PEM columns * = *p* < 0.01 or ** = *p* < 0.001 for post hoc analysis vs. controls. In the ME/CFS NoPEM or PEM columns † = *p* < 0.01 for post hoc analysis of NoPEM vs. PEM.

**Table 3:** PEM 7-day severity and 12-month frequency score correlate with biochemistry in the Serum urine and fecal metabolomes.

Serum	7-Day PEM All Subjects	7-Day PEM ME/CFS Subjects	12-Month PEM All Subjects	12-Month PEM ME/CFS Subjects
Phenylalanine	−0.40 **	−0.11	−0.42 **	−0.08
Hypoxanthine	−0.35 *	+0.25	−0.43 **	+0.21
Lactate	−0.33 *	+0.13	−0.37 *	+0.18
Threonine	−0.31 *	−0.13	−0.25	+0.07
Glucose	+0.31 *	−0.09	+0.38 **	+0.02
Urine				
Total Metabolite	+0.10	+0.38 *	−0.02	+0.18
Mannitol	−0.01	+0.43 *	−0.15	+0.20
Serine	−0.07	+0.42 *	−0.22	+0.17
Acetate	−0.18	+0.41 *	−0.32 *	+0.21
*p*-Methylhistidine	+0.08	+0.40 *	−0.14	−0.02
Glucose	+0.02	+0.37 *	−0.09	+0.23
Urine %				
Urea%	−0.42 **	−0.24	−0.37 **	−0.04
Pyruvate%	−0.35 *	+0.06	−0.37 **	0.13
Tryptophan%	−0.32 *	−0.28	−0.20	0.06
Malonate%	−0.32 *	−0.37 *	−0.18	0.05
Acetate%	−0.30 *	+0.06	−0.35 *	−0.01
Fecal %				
Uracil	+0.04	+0.46 **	−0.09	0.27

Statistical method: Spearman Rank correlation. Multiplicity correction *p* < 0.01, * = *p* < 0.01 or ** = *p* < 0.001.

**Table 4:** Assessment of Purine metabolite changes in serum and urine in the PEM, NoPEM and control subjects.

Metabolite	Control	NoPEM	PEM
Serum Hypoxanthine	15.7 ± 12.2 **	3.6 ± 1.4 **	6.6 ± 8.2 **
% Serum Hypoxanthine	0.55 ± 0.39% **	0.14 ± 0.05% **	0.24 ± 0.25% **
Urine Hypoxanthine	14.9 ± 6.7 *	7.6 ± 4.0*	14.3 ± 11.3
%Urine Hypoxanthine	0.26 ± 0.13% *	0.17 ± 0.04%	0.21 ± 0.10%
Urine Allantoin	32.8 ± 19.0	36.0 ± 13.5	43.7 ± 25.1
% Urine Allantoin	0.53 ± 0.28% *	0.96 ± 0.33% *	0.78 ± 0.53%
Serum Urate	0.28 ± 0.03	0.28 ± 0.06	0.29 ± 0.09
Serum Purine Ring Precursors	138.6 ± 32.8	117.9 ± 23.9	130.2 ± 29.8
Ratios			
Serum Hypoxanthine: Urine Hypoxanthine	1.3 ± 1.6	0.7 ± 0.6	0.9 ± 1.4
Serum Hypoxanthine: Urate	74.2 ± 65.3 **	13.6 ± 6.2 **	21.6 ± 26.3 **
Serum Hypoxanthine: Urine Allantoin	0.75 ± 1.26	0.11 ± 0.05	0.34 ± 0.09
Urine Allantoin: Serum Urate	135.5 ± 71.7	134.2 ± 57.1	162.5 ± 96.2

Statistical method: ANOVA. In control column * = *p* < 0.05 or ** = *p* < 0.001 for the ANOVA. In the ME/CFS NoPEM or PEM columns * = *p* < 0.05 or ** = *p* < 0.001 for post hoc analysis vs. controls.

## Supplementary Materials

The following are available online at https://www.mdpi.com/2075-4418/9/3/70/s1.

## Abbreviations

BMP	Bone Morphogenic Protein
HDAC	Histone Deacetylase
ME/CFS	FS Myalgic encephalomyelitis/Chronic Fatigue Syndrome
PEM	Post-Exertional Malaise
SMAD	Transforming Growth Factor-Beta Signaling Proteins
TGF-β	Transforming Growth Factor-Beta
TCA	tricarboxylic acid cycle
