# Supplementary material for: Post-Exertional Malaise Is Associated with Hypermetabolism, Hypoacetylation and Purine Metabolism Deregulation in ME/CFS Cases
Source: Diagnostics (Basel). 2019 Jul 4;9(3):70. doi: 10.3390/diagnostics9030070 (PMC6787670; doi:10.3390/diagnostics9030070)

Supplementary data. Post Exertional Malaise, Hypoacetylation and Purine metabolism deregulation in ME/CFS Cases. Neil R. McGregor[1] PhD. Christopher W. Armstrong[2] PhD, Don Lewis MBBS, Paul Gooley PhD.

Table S1. HDAC binding sites to the 20 upregulated genes found in Whistler et al. 2005 and the binding sites in the glycolysis rate limiting genes. The genes are regulated by both HDAC1 and HDAC2 but also Bone Morphogenic Protein Receptor related transcription factors SMAD1, SMAD4 and SMAD5.

**Glycolysis Genes**	**HDAC1**	**HDAC2**	**SMAD1**	**SMAD4**	**SMAD5**
HK1	1	1	1	1	1
PFKM	1	1	1	1	1
PKM	1	1	1	1	1
PKLR	1	1	1	1	1
PDHA1	1	0	1	0	1
PDHX	1	1	1	1	1
PDHB	1	1	1	1	1
PDP1	1	1	0	0	0
PDK1	1	1	1	1	1
PDK2	1	1	1	1	1
PDK4	1	1	0	1	0
G6PD	1	1	1	1	1
CPS1	1	0	0	1	1
**Whistler et al 2005**					
ZNF124	1	1	1	1	1
PAX8	1	1	1	1	1
PDHX	1	1	1	1	1
BRCA2	1	0	1	0	1
TNK1	1	1	1	1	1
SYN3	1	1	1	1	0
STX11	1	1	1	1	1
DYSF	1	1	0	1	0
USO1	1	1	1	1	1
TMEFF1	1	1	1	1	1
IL18RAP	1	1	1	1	1
CADPS	0	1	0	1	0
PIP5K1B	1	1	1	1	1
IL1RL2	1	1	1	1	1
**HIST1H4H**	1	1	1	1	1
AKAP1	1	1	0	1	1
TAF15	1	1	1	1	1
**HIST2H2BE**	1	1	1	1	1
SCARF1	1	1	1	1	1
STAM	1	1	1	1	1

0= No binding site present, 1= binding site present,

Figure S1. Canonical analysis of PEM, NoPEM and Control subgroupings using combined Serum, Urine and Fecal metabolome data.

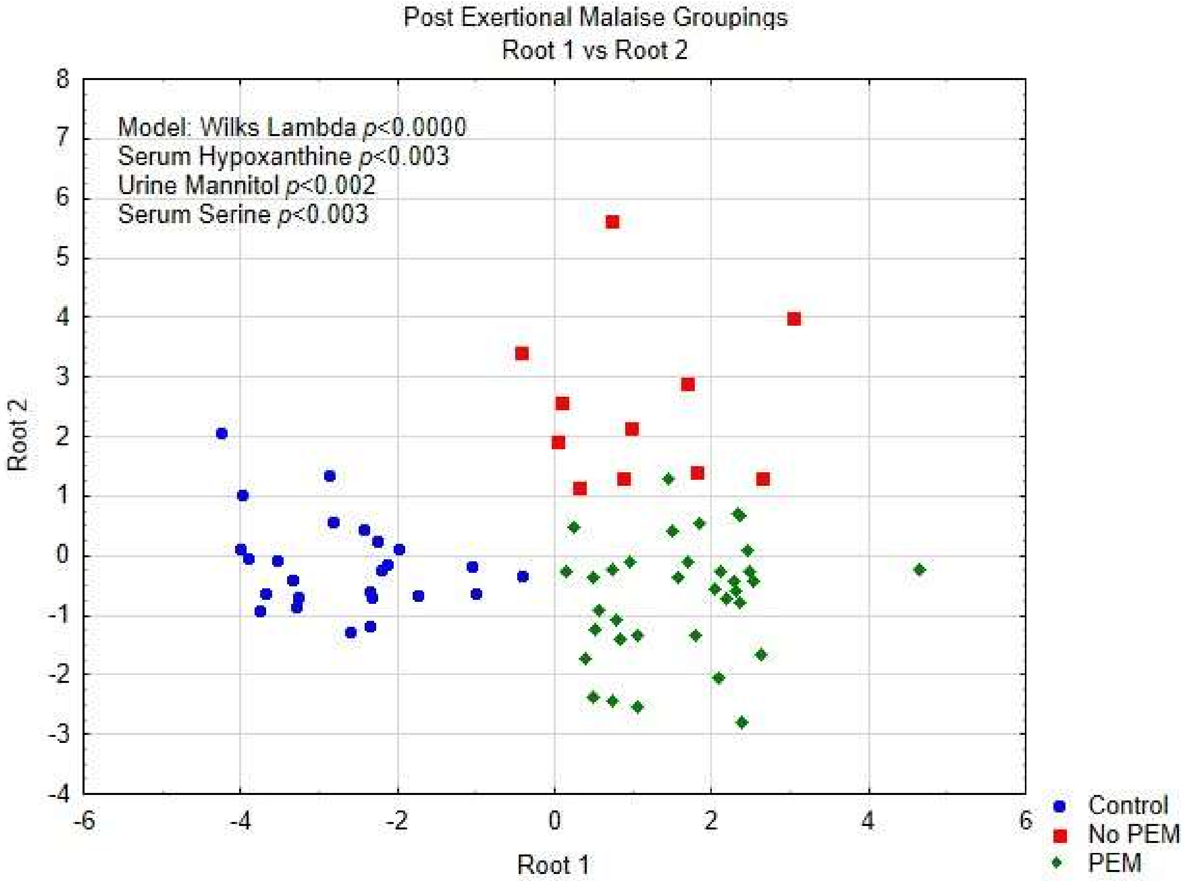

Comparison of metabolomes between ME/CFS and Control subjects to match PEM symptom reporting. Discriminant function analyses.

Serum: Wilks' Lambda: 0.16 approx. F (60,78) = 1.90 $p<0.004$.
Significant metabolites. Serine $p<0.003$, Glucose $p<0.008$.

Urine: Wilks' Lambda: 0.16 approx. F (62,76) = 1.79 $p<0.008$.

Fecal: Wilks' Lambda: 0.30 approx. F (48,90) = 1.55 $p<0.04$.
Significant metabolites. Glutamate $p<0.03$, Malonate $p<0.04$.

Combined Data: Forward Stepwise: Wilks' Lambda: 0.04 approx. F (68,70) = 4.10 $p<0.0000$
Significant metabolites. 1. Serum Hypoxanthine $p<0.003$, 2. Urine Mannitol $p<0.002$, 3. Serum Serine. $p<0.003$, 4. Serum Threonine. $p<0.003$, 5. Serum Glucose. $p<0.03$.

Metabolomes in order of significance in predicting the group differences: Serum > Urine > Faecal.

Figure S2. Serum Purine Ring Precursors vs. Hypoxanthine

Control: r = 0.61 $p<0.001$

ME/CFS : r = 0.66 $p<0.001$

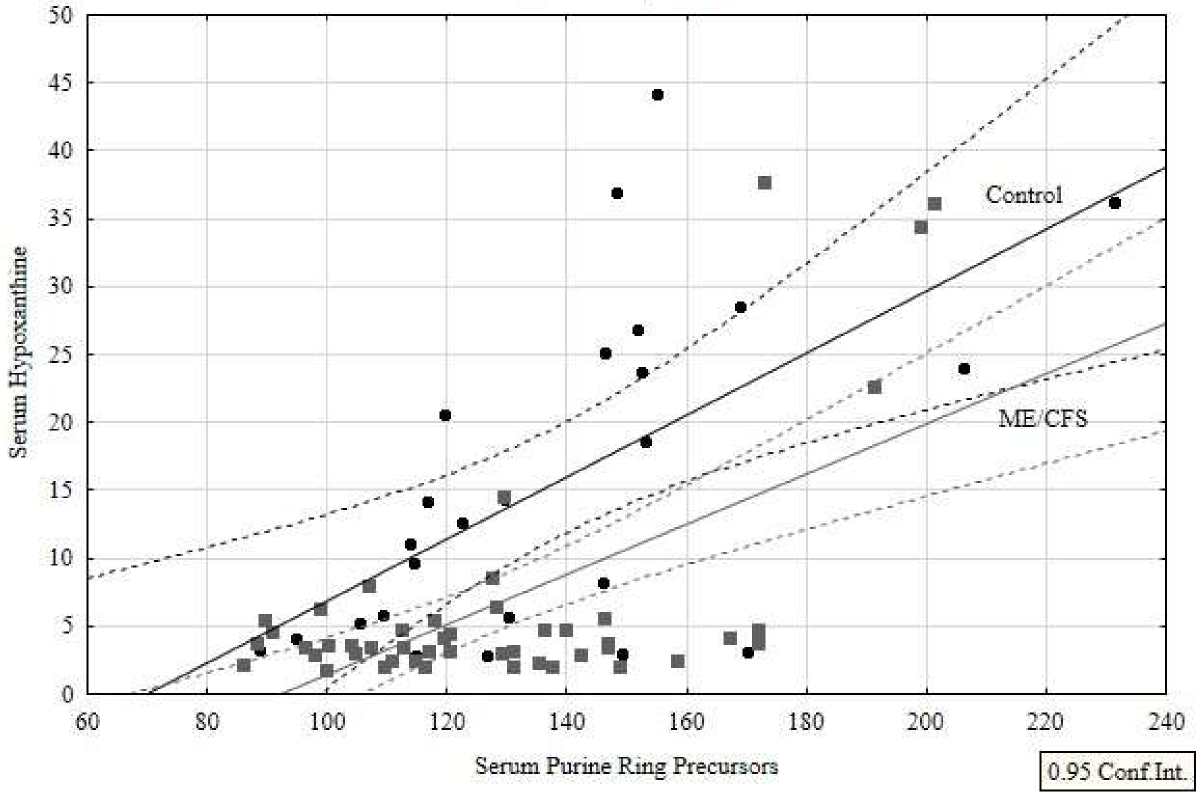

Supplement: Supplementary file 1 [file diagnostics-09-00070-s001.pdf]
